# Solitary scalp metastasis – a rare presentation of hepatocellular carcinoma

**DOI:** 10.1186/s13022-015-0013-2

**Published:** 2015-06-09

**Authors:** Duminda Subasinghe, Chathuranga Tisara Keppetiyagama, Hemantha Sudasinghe, Saman Wadanamby, Niranthi Perera, Sivasuriya Sivaganesh

**Affiliations:** University Surgical Unit, The National Hospital of Sri Lanka, Colombo, Sri Lanka; Department of Neurosurgery, The National Hospital of Sri Lanka, Colombo, Sri Lanka; Department of Pathology, Faculty of Medicine, University of Colombo, Colombo, Sri Lanka; Department of Surgery, Faculty of Medicine, Kynsey Road, Colombo 8, Colombo, Sri, Lanka

**Keywords:** Hepatocellular carcinoma, Skull metastasis

## Abstract

**Introduction:**

Hepatocellular carcinoma (HCC) is among the commonest cancers in the world. Metastasis is one of the most significant factors affecting prognosis. Common sites of extrahepatic metastases include lungs, regional lymph nodes and less commonly bone.

**Case presentation:**

A 56-year-old male presented with a painless occipital scalp lump of three months duration, with recent rapid enlargement. His skull x-ray showed a lytic lesion over occipital bone and the contrast CT scan of the brain showed a scalp mass with destruction of the adjacent skull. Core biopsy of the lesion revealed a metastatic deposit from a hepatocellular carcinoma.

**Conclusion:**

Primary presentation with skeletal metastases are rare in HCC with only a few reported cases. Here we report a case of HCC presenting as a solitary scalp lump.

## Background

Hepatocellular carcinoma is one of the most prevalent cancers worldwide, especially in Asia and Africa [1]. The high incidence of HCC in Asia is related to the increased prevalence of chronic viral hepatitis B [2]. HCC commonly metastasises to regional lymph nodes and lungs [3], and less commonly to bone. Cutaneous metastases are extremely rare with only few reports in the literature [4]. Though indicative of a poor prognosis, aggressive early identification and treatment can help improve the quality of life in these patients [4]. Here we describe a patient without previous liver disease presenting with a solitary skull deposit as the first manifestation of HCC.

## Case presentation

**Fig. 1:**
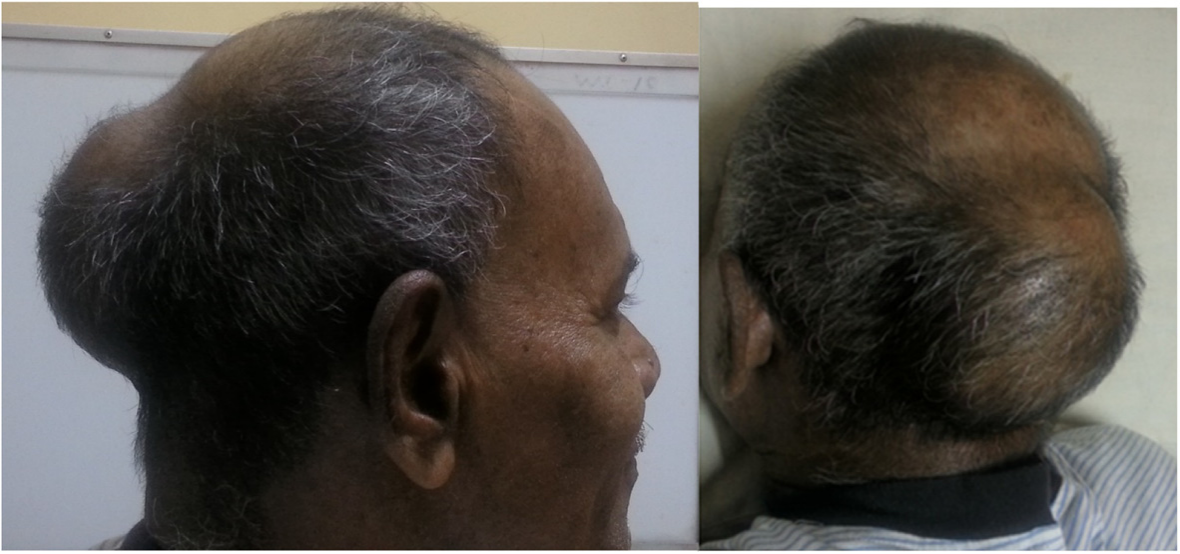
Occipital scalp lump. **a**-lateral view, **b**-posterior view

**Fig. 2:**
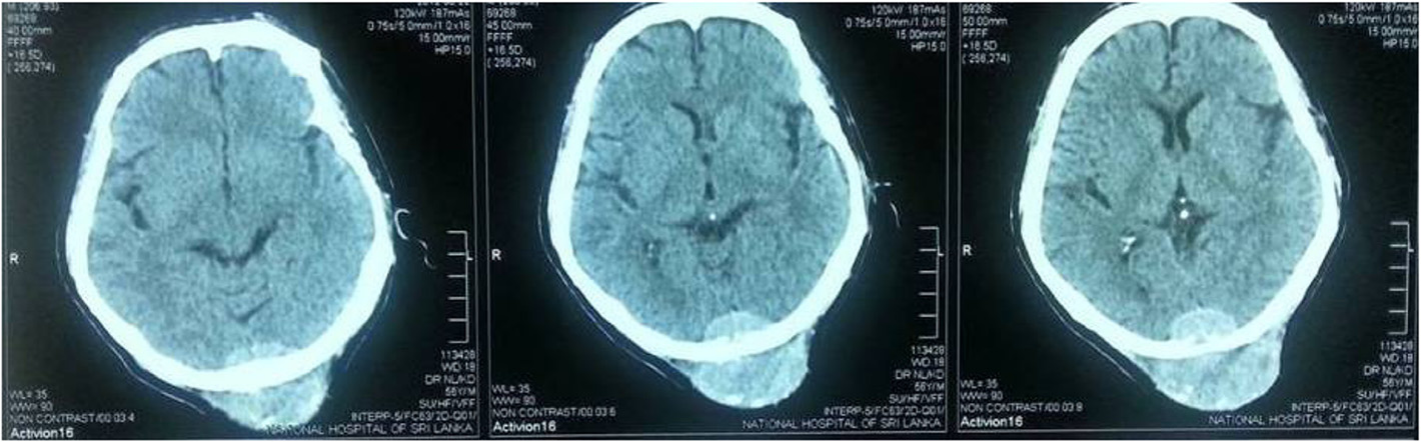
Contrast CT brain showing skull deposit with extra and intracranial extension

**Fig. 3:**
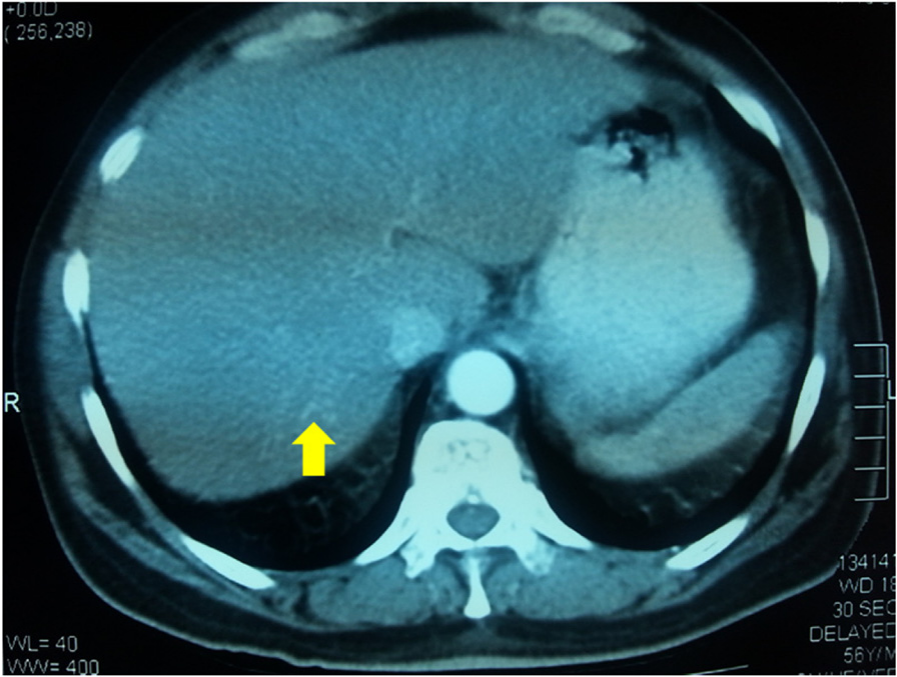
Contrast CT abdomen showing hepatoma in segment VI

**Fig. 4:**
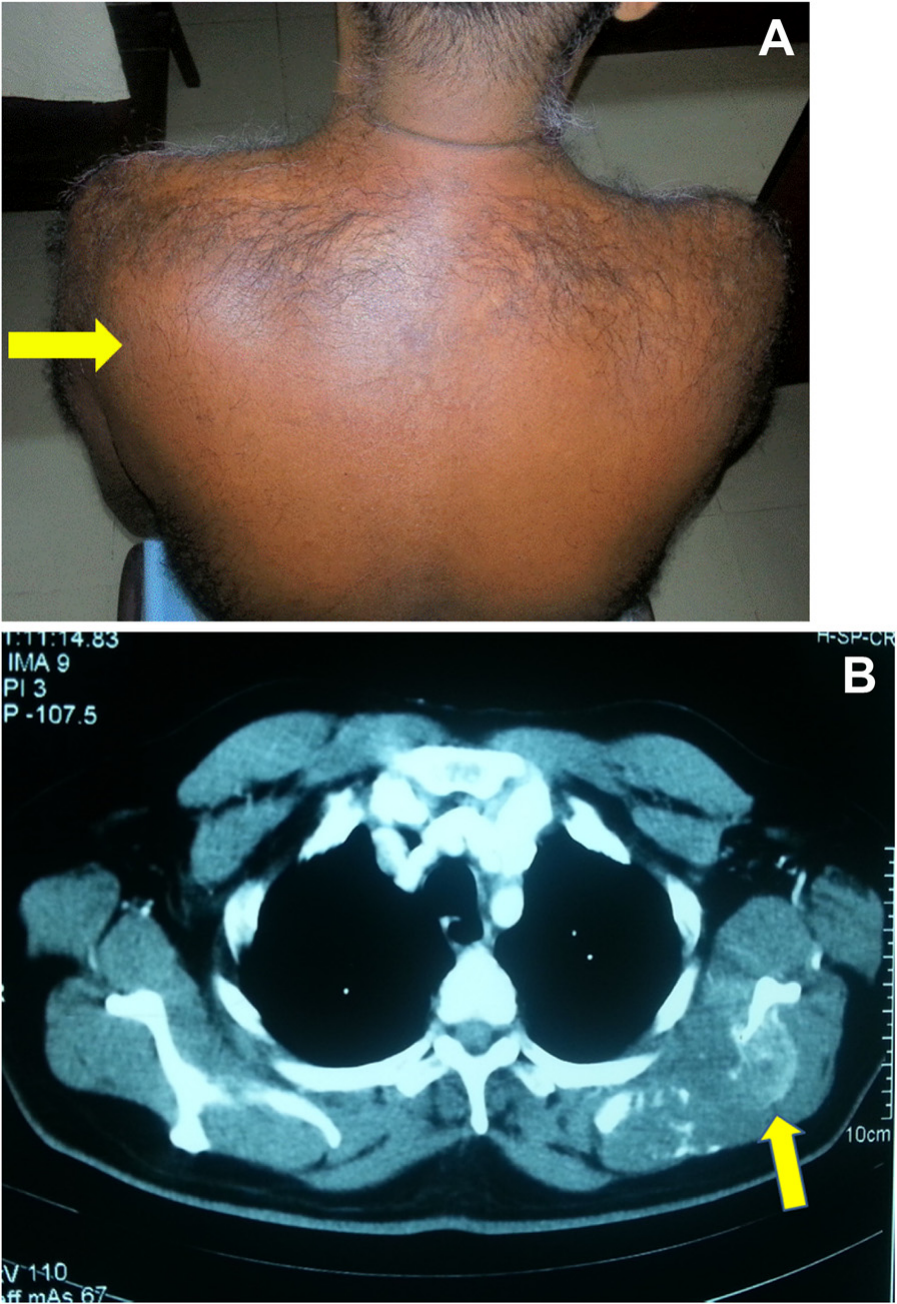
**a** Swelling over left scapular region. **b** Arrow indicates metastatic bone destruction of left scapula

A 56-year-old male presented with a painless occipital scalp lump of three months duration, with recent rapid enlargement. There was no history of head injury and no associated headache or vomiting. Despite a history of significant alcohol consumption for over 15 years there was no history of jaundice or prior liver disease. He was free of upper and lower gastrointestinal symptoms, his appetite was good and there was no weight loss. He was a diabetic for nine years on oral hypoglycaemic therapy. Examination revealed a non-tender, hemispherical, subcutaneous lump over the occipital region (Fig. 1a and b) approximately 10 cm in diameter. The lump was soft to firm in consistency, non-pulsatile and with no demonstrable cough impulse. His general and systemic examination including the abdomen was otherwise unremarkable with no stigmata of chronic liver disease and no focal neurological signs. His routine blood biochemistry including liver function tests was normal. His skull x-ray showed a lytic lesion of the occipital bone. The contrast CT scan of the brain showed a scalp mass with destruction of the adjacent skull vault and intracranial extension but (Fig. 2) no penetration of the meninges. Core biopsy of the lesion revealed a metastatic deposit from a hepatocellular carcinoma. This was confirmed by immunohistochemical analysis demonstrating positivity for alpha fetoproteins and hepar 1. Subsequent triple phase CT scan of the abdomen (Fig. 3) showed a 3.5 cm diameter lesion in segment VI of the liver with characteristic features of HCC but no radiological evidence of cirrhosis. His alpha fetoprotein titre was elevated > 392 KU/l (normal range 0–10 KU/l). Screening was negative for hepatitis B. His chest x-ray was normal. The patient underwent palliative excision of the scalp deposit to prevent cerebral complications. During surgery the dura mater was found to be intact. The post-operative period was uncomplicated. He subsequently underwent radiofrequency ablation (RFA) of the primary in the liver. During follow up, he developed a painful soft tissue swelling over the left scapular region (Fig. 4a). CT scan revealed a subcutaneous mass with destruction of the left scapula (Fig. 4b), confirmed by FNAC to be metastatic. He was referred to the oncologist for palliative control of this lesion.

## Discussion

Extrahepatic metastases generally occur late in in the course of HCC because most patients succumb to liver failure resulting from of liver parenchymal invasion and replacement by the tumor. Common sites of extrahepatic metastases in HCC include the lungs and to a lesser extent the bone [5]. Skull metastasis is rare with a reported incidence of 0.5-1.6 % [5–7] and generally occurs in advanced systemic disease. Cutaneous metastases of solid organ malignancies are rare and commonly present after diagnosis of the primary. The incidence of cutaneous metastases in malignancies of the lung, colon, rectum, kidney, bladder and ovary is 3.4 % - 4.0 % [8]. The majority of subcutaneous metastases of HCC are iatrogenic, occurring in the needle tract of FNAC or in surgical wounds [9, 10]. There is a paucity of reports on spontaneous subcutaneous deposits possibly because they predominantly present in patients with end stage disease. However, according to Amador *et al.* it could be the sole and initial presenting feature of HCC [11].

Metastases eroding the vault of the skull may be complicated by epidural haematomas and intratumoral haemorrhage resulting in neurological sequelae [12, 13]. Excision of the deposit in this patient was done in order to prevent such a complication. RFA of the liver lesion was undertaken since no evidence of further secondary disease was evident at the time (*i.e.* prior to the identification of the scapular deposit). This is a report of a rare presentation of HCC as a solitary skull metastasis with an asymptomatic primary. Aggressive treatment of local disease and the primary prevents rapid deterioration due to complications and enables a better quality of life in this stage of HCC with an otherwise dismal prognosis.

### Consent

Written informed consent was obtained from the patient for publication of this case report and any accompanying images. A copy of the written consent is available for review by the Editor-in-Chief of this journal.

## Abbreviations

HCC: Hepatocellular carcinoma
CT: Computed tomography
RFA: Radiofrequency ablation
FNAC: Fine needle aspiration cytology
